# Effectiveness of combination of Mini-and Microsatellite loci to sub-type *Mycobacterium avium *subsp. *paratuberculosis *Italian type C isolates

**DOI:** 10.1186/1746-6148-7-54

**Published:** 2011-09-19

**Authors:** Matteo Ricchi, Gianluca Barbieri, Roberta Taddei, Gian L Belletti, Elena Carra, Giuliana Cammi, Chiara A Garbarino, Norma Arrigoni

**Affiliations:** 1 Istituto Zooprofilattico Sperimentale della Lombardia e dell'Emilia Romagna. National Reference Centre for Paratuberculosis. Strada Faggiola 1, 29027 Gariga di Podenzano (Piacenza). Italy; 2 Istituto Zooprofilattico Sperimentale della Lombardia e dell'Emilia Romagna. Diagnostic Section of Forlì, Italy; 3 Istituto Zooprofilattico Sperimentale della Lombardia e dell'Emilia Romagna. Diagnostic Section of Modena, Italy

## Abstract

**Background:**

*Mycobacterium avium *subsp. *paratuberculosis *(Map) is the etiological agent of paratuberculosis. The aim of our study was to combine Mini-and Microsatellite loci analysis in order to explore the effectiveness of this sub-typing method in a group of Map isolates. For this purpose, 84 Italian Type C Map isolates, each from a different cattle herd, were submitted to MIRU-Variable-Number Tandem-Repeats (VNTRs) typing and Short Sequence repeats (SSRs) sequencing. Moreover, the method was used to analyse the variability inside 10 herds (from three to 50 isolates per herd).

**Results:**

The molecular sub-typing, carried out using three SSR and 10 MIRU-VNTR loci, differentiated the 84 isolates into 33 clusters, reaching a Simpson's Discriminatory Index (SID) value of 0.952 (0.933 to 0.972, 95% confidence intervals). Among all considered loci, six (SSR2, MIRU2, SSR1, SSR8, VNTR3527 and VNTR1067) showed relevant allelic variability. Thirty-eight% of the isolates were clustered into four genotypes, differing from each other for the SSR2 locus. The other isolates, characterised by differences in two or more loci, were spread among the rest of the clusters. The intra-herd analysis revealed more than one genotype in most herds with a similar distribution of clusters.

**Conclusions:**

Our results revealed the advantage of using both Mini-and Microsatellite approaches for successfully discriminating among Map Type C isolates from the same geographic area, host species and herd. These data suggest that the combination of loci here proposed could be a useful molecular tool for regional epidemiological studies.

## Background

*Mycobacterium avium *subsp. *paratuberculosis *(Map) is the etiological agent of paratuberculosis in cattle. This disease can affect many ruminant species and other wildlife animals [1].

Paratuberculosis is characterized in cows by severe gastroenteritis, diarrhoea, weight loss, reduced milk production and premature culling, leading to economic losses [2]. Moreover, although still debated, some data suggest a possible involvement of Map in Crohn's disease [3].

The knowledge of the causative agent of Paratuberculosis, both in terms of epidemiology and biodiversity within different strains, could be useful, especially in case of new outbreaks or in following Map's diffusion pathway.

According to their genetic differences [1,4], Map strains have been classified into four groups. However, this classification only partially reflects their host-specificity. Type S (sheep), also designed as Type I, seems to be largely prevalent in sheep, while type C (cattle), also known as Type II, represents the type most widely recovered from various hosts and sources. Type B (bison) is considered a subtype of Type C. In addition, a sub-type of Type I, designed as Type III, has been reported [1,4].

Beyond the previously described classification, many methods have been proposed for Map sub-typing (reviewed in [4]); in particular, those based on the amplification of repetitive-element loci, at present, are considered the emerging Map sub-typing techniques [4].

The repetitive-element loci herein analysed, both Mini-and Microsatellite loci (for the definition see [5]), have been previously described [6-9] and their discriminatory power has been evaluated [10-13].

The aim of this study was to explore the effectiveness of this technique in isolates from the same country and host species. For this purpose, we evaluated a combination of Mini-and Microsatellite loci in a panel of 84 type C Map isolates, each recovered from a different Italian cattle herd. In order to further investigate the effectiveness of this technique, the proposed method was also applied to 98 Map isolates originated from 10 herds (from three to 50 isolates recovered from each single herd).

## Results

All the isolates were classified as Type C, showing a PCR product of 310 bp (DMC-PCR) and the typical Type C pattern in IS1311 PCR-REA (fragments 67 bp, 218 bp, 285 bp and 323 bp) (data not shown).

### Minisatellite loci

Ten Minisatellite loci were considered for the calculation of the Simpson's Index of Diversity (SID). Locus MIRU2 showed the highest allelic diversity (*h*) (Additional File 1, Table S1), followed by loci VNTR1067, VNTR3527, VNTR25, VNTR7, VNTR32, MIRU3 and VNTR47. Finally, loci MIRU1 and VNTR3 did not show any allelic diversity in our isolates.

Taken together, Minisatellite loci revealed 11 clusters (Table 1), with a SID value of 0.686 (0.597-0.775, CI 95%). Two main genotypes (types MV 10 and MV 4) clustered the 51% and 21% of the total isolates (Table 1). Type MV 9 clustered the 8% and type MV 5 and type MV 3 the 6% and the 5%, respectively. These five genotypes differed from each other for three loci: MIRU2, VNTR1067 and VNTR3527.

**Table 1:** MIRU-VNTR pattern of the 84 Type C isolates coming from different herds.

			No. of copies MIRU-VNTR									
**MIRU-VNTR types**	**No. of isolates**	**%**		**MIRU**^**a**^					**VNTR**^**b**^			

			1	2	3	25	32	3	7	47	1067	3527
MV 1	1	1.2	3	7	3	3	8	2	2	3	2	1
MV 2	1	1.2	3	9	5	3	8	2	1	3	2	2
MV 3	4	4.8	3	9	5	3	8	2	2	3	3	2
MV 4	18	21.4	3	9	5	3	8	2	2	3	2	2
MV 5	5	5.9	3	9	5	3	8	2	2	3	2	1
MV 6	1	1.2	3	7	5	3	8	2	1	3	2	2
MV 7	1	1.2	3	7	5	3	8	2	3	3	2	2
MV 8	2	2.4	3	7	5	3	8	2	2	3	1	2
MV 9	7	8.3	3	7	5	3	8	2	2	3	2	1
MV 10	43	51.2	3	7	5	3	8	2	2	3	2	2
MV 11	1	1.2	3	5	5	5	6	2	2	2	2	2

^a ^MIRU loci according to Bull et al. [6].

^b ^VNTR-MIRU according to Overduin et al. [8] for loci 1067 and 3527 and according to Thibault et al. [9] for loci 25, 32, 3, 7, 47.

### Microsatellite loci

Among the 11 SSR loci proposed by Amonsin [7], three loci (loci SSR1, 2 and 8) were selected for the analysis. The selection was made according to previous evidence indicating that these loci showed the highest allelic diversities [7,12,14-17]. These observations have been preliminarily confirmed analysing a set of 10 Italian isolates [18].

The locus SSR1 showed five alleles (Table 2), while the loci SSR2 and SSR8 showed four and three alleles respectively. Overall, the analysis of SSRs loci grouped the isolates into 15 clusters, with a SID of 0.840 (0.799-0.881, CI 95%). Four main genotypes (types S 1, S 2, S 3 and S 4) clustered the 75% of the total, differentiating each other only for the locus SSR2.

**Table 2:** SSR pattern of the 84 Type C isolates coming from different herds

				**SSR**^**a**^	
**SSR types**	**No. of isolates**	**%**	**1**	**2**	**8**

S 1	6	7.1	7	9	4
S 2	23	27.4	7	10	4
S 3	18	21.4	7	11	4
S 4	16	19.0	7	> 11	4
S 5	1	1.2	8	> 11	5
S 6	1	1.2	8	9	3
S 7	2	2.4	8	10	4
S 8	1	1.2	9	11	4
S 9	2	2.4	10	10	5
S 10	1	1.2	> 11	> 11	5
S 11	1	1.2	> 11	10	4
S 12	5	6.0	> 11	10	5
S 13	3	3.6	> 11	11	5
S 14	2	2.4	> 11	> 11	5
S 15	2	2.4	> 11	> 11	4

^a ^SSR according to Amonsin et al. [7].

### Combination of both Mini-and Microsatellite loci

**Figure 1:**
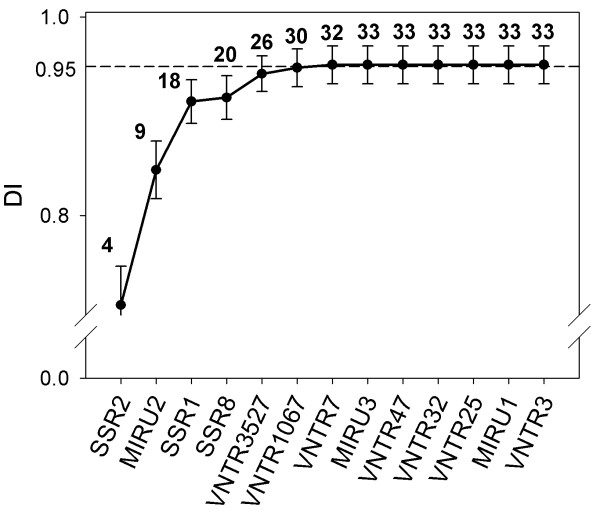
**The 13 loci were sorted by allelic diversity, starting from the highest value and adding each locus to the sub-typing analysis**. All the obtained combinations were analysed separately, in order to evaluate the SID trend. Confidence intervals of 95% were also showed. The numbers over the curve indicates the clusters obtained with the specific loci combination. Note that the SID value increased until locus MIRU3 (0.952).

A total of 33 different clusters originated from the combination of the 13 loci, both Mini-and Microsatellites (Figure 1 and Table 3). The final SID was 0.952, ranging from 0.933 to 0.972 (CI 95%). Three main genotypes resulted from the analysis of the combination between MIRU-VNTR and SSR typing (types MVS 25, MVS 26 and MVS 27, Table 3). These three genotypes showed always the same MIRU-VNTR type (type MV 10) but differed for the SSR type at SSR2 locus level. The types MVS 26 and MVS 27 seem to be the most recovered genotypes in the northern Italy, while the type MVS 25 was present even in the centre and south of the country (Table 3).

**Table 3:** MIRU-VNTR/SSR pattern of the 84 Type C isolates coming from different herds.

	MIRU-VNTR/SSR types	No. of isolates	%	MIRU-VNTR **types**^**a**^	SSR **types**^**b**^	**Herd No.**^**c**^
	MVS 1	1	1.2	MV 1	S 4	37
	MVS 2	1	1.2	MV 2	S 3	18
	MVS 3	2	2.4	MV 3	S 2	64, 69
	MVS 4	2	2.4	MV 3	S 3	15, 61
	MVS 5	3	3.6	MV 4	S 1	2, 23, 66
	MVS 6	4	4.8	MV 4	S 2	54, 55, 82, 84
	MVS 7	3	3.6	MV 4	S 3	**8**, 49, 59
	MVS 8	3	3.6	MV 4	S 4	5, 14, 21
	MVS 9	1	1.2	MV 4	S 6	81
	MVS 10	2	2.4	MV 4	S 7	10, 71
	MVS 11	1	1.2	MV 4	S 8	39
	MVS 12	1	1.2	MV 4	S 11	1
	MVS 13	3	3.6	MV 5	S 2	17, 25, 72
	MVS 14	2	2.4	MV 5	S 4	29, 63
	MVS 15	1	1.2	MV 6	S 15	47
	MVS 16	1	1.2	MV 7	S 4	28
	MVS 17	1	1.2	MV 8	S 12	65
	MVS 18	1	1.2	MV 8	S 14	36
	MVS 19	3	3.6	MV 9	S 2	41, **45**, 51
	MVS 20	1	1.2	MV 9	S 3	9
	MVS 21	1	1.2	MV 9	S 4	12
	MVS 22	1	1.2	MV 9	S 5	62
	MVS 23	1	1.2	MV 9	S 15	83
	MVS 24	3	3.6	MV 10	S 1	31, 42, 67
	MVS 25	10	11.9	MV 10	S 2	24, 27, 34, 40, 48, 58, 60, **73**, 74, 76
	MVS 26	11	13.1	MV 10	S 3	6, 13, 16, 30, 35, 43, 44, **57**, 68, 77, 79
	MVS 27	8	9.5	MV 10	S 4	4, 7, 22, 32, 53, 56, 70, 80
	MVS 28	2	2.4	MV 10	S 9	20, 26
	MVS 29	1	1.2	MV 10	S 10	50
	MVS 30	4	4.8	MV 10	S 12	11, 46, 75, 78
	MVS 31	3	3.6	MV 10	S 13	19, 38, 52
	MVS 32	1	1.2	MV 10	14	33
	MVS 33	1	1.2	MV 11	S 2	3

**SID**	**0.933-0.972**			**0.597-0.775**	**0.799-0.881**	

^a ^according to Table 1

^b ^according to Table 2

^c ^in bold and underlined are indicated herds in the centre and south of the country, respectively, while the rest of the herds are placed in the north of Italy.

Considering the three loci with the highest *h *(SSR2, MIRU2 and SSR1), we obtained 18 clusters, with a SID value of 0.915 (0.893-0.937) (Figure 1). Adding more loci to the SID calculation, the slope of the graph increased slowly, finally reaching the plateau phase after the locus MIRU3.

The proposed method was then used to test the inside-herd variability. Even after this analysis (Additional File 2, Table S2), the genotypes most frequently found were MVS 26 and MVS 27, while others, like types MVS 31 and MVS 32, seem to be confined to only two herds placed in two adjacent regions (Lombardy and Emilia Romagna, respectively). Moreover, this investigation allowed finding out eight further genotypes not detected with the previous analysis.

Three or more distinct genotypes, differing from each other for both Mini-and Microsatellite loci, were isolated from three herds placed in the same area (herds 31, 32 and 33), while in four herds, two (herds 43, 59 and 79) or three different genotypes (herd 82), diverging each other only for the SSR2 locus, were recovered (Additional File 2, Table S2).

## Discussion

This study was mainly conducted to evaluate the effectiveness of a panel of selected Mini-and Microsatellite loci for sub-typing closely related isolates. The 84 isolates included in this study have not been previously sub-typed with any other method and were identified as Type C. Most of the isolates analysed were from two northern Italian regions (Lombardy and Emilia Romagna), where about the 60% of the National dairy farms is located. Few isolates derived from other northern regions (Piedmont and Veneto), while the rest of the isolates came from herds located in the centre and south of Italy. As a result, the sampling was not representative of the whole country, so epidemiological conclusions about the distribution of the different genotypes could not be drawn. Moreover, it is very plausible the existence of links related to cattle trading which can further complicate the epidemiological analysis.

Considering the 84 isolates from different herds, despite the above mentioned limits, type MVS 25 profile was recovered in the whole country, while those containing types MVS 26 and MVS 27 seem to be confined in the northern Italy. These genotypes showed the most frequent MIRU-VNR pattern profile and differed for the locus SSR2. In a recent paper, Douarre et al. [13] reported similar results for 38 type C isolates from Ireland. These authors concluded that the analysis of Minisatellite loci could be useful for producing broad epidemiological data, especially in terms of establishing the interrelatedness of the isolates, while Microsatellite loci seem to be more informative producing unique genetic fingerprints.

Even the intra-herd analysis confirmed that the types most frequently found were MVS 26 and MVS 27, while others types seem to be limited to some herds (e.g. type MVS 32). As recently reported [19], we noticed that herds characterised by an elevated turnover of cows showed many sub-types of Map (i.e. herds no. 31, 32 and 33), which differed each other for both Mini-and Microsatellite loci (Additional File 2, Table S2). Interestingly, the herds 57 is the only herd in the south of the country where type MVS 26 was recovered, suggesting, as a possible route of infection, the introduction of infected animals from the northern Italy.

More in general, the presence of different types of Map within the same herd might suggest i) multiple infection due to the introduction of infected animals or ii) divergence of the isolates caused by locus instability.

Regarding this last hypothesis, as inferred from previous reports [7,11-13,16,17], locus SSR2 showed a very high variability. The stability of the SSRs loci has been demonstrated "*in vitro*" by testing three strains after ten subcultures [17], but no data are available about the *"in vivo" *stability. Nevertheless, in *M. avium 104 *genome, the locus SSR2 is localised in an intergenic region between "*rph*" and "*rdgB*" genes, while the same region on Map K10 does not seem to code for any protein, suggesting the hypothesis that this locus could be a potential hot spot for mutation occurrence.

Loci SSR1 and SSR8 showed lower variability in comparison with locus SSR2. The two most frequently found alleles for the locus SSR1 had seven and more than 11 repeats (76% and 15% of the isolates, respectively). Similar results were achieved for locus SSR8 with the alleles containing four and five repeats (70% and 28%, respectively). For this latter locus we found one isolate containing the allele with three repeats, which has been observed only in Map type S strains [7,11-13,16,17].

The DNA region target of MIRU3 locus seems to be related to some components of the regulatory system involved in oxidative response in other mycobacteria [15]. In our isolates the predominant allele carried five repetitions, a variant previously associated with a higher degree of virulence compared to those with three repeats (i.e vaccine strain 316F). However, we found two isolates with this last allele (types MVS 1 and MVS G2, see Table 3 and Additional File 2, Table S2) in two farms placed in adjacent provinces of the northern Italy, confirming that, although rare, it is circulating even in field strains [9,10].

Guidelines for validation and application of typing methods for epidemiology [20] proposed that the discriminatory index value for the evaluation of epidemiological data should be upper than 0.95. In our study, the analysis of 13 loci enabled a SID of 0.952. However, some authors considered even 0.90 as an acceptable cut-off value, because Map is an organism characterised by limited genetic diversity [1]. In this regard, an exceeding result (0.915, from 0.893 to 0.937) can be reached considering only the three loci with the highest allelic diversity (SSR2, MIRU2 and SSR1) (Figure 1). Accordingly, these loci could be considered the first choice for sub-typing field isolates.

Finally, our data indicated that the use of Minisatellite loci alone did not seem appropriate to reach good discriminatory indexes in homogeneous group of isolates (SID 0.686). These findings suggest its application should be coupled with other techniques (i.e. PFGE or RFLP [1,10,16]) or eventually used to sub-type among different Map types [15].

## Conclusions

Our data confirm the combined use of Mini-and Microsatellite loci as a suitable tool to sub-type Map, even in isolates recovered from the same host species (cattle), originating from the same country (Italy) and belonging to the same type (Type C). MIRU-VNTR loci analysis is a robust and simple technique, but, in order to reach appropriate discriminatory indexes among homogeneous isolates, it should be associated with the analysis of SSR loci or other techniques.

Finally, these data suggest that the combination of loci here proposed, coupled with other epidemiological information (i.e. animal trading), could be a useful molecular tool for both national and regional epidemiological studies.

## Methods

### Isolates collection

Eighty-four Map isolates, each from a different herd, were enrolled in this study. The herds were primarily located in two northern Italian regions, Lombardy and Emilia Romagna. When more than one isolate was recovered from the same herd, the one included in the analysis was randomly selected.

For the evaluation of the inside-herd diversity, we considered even 98 isolates recovered from 10 herds (ranging from three to 50 isolates from each herd). Herds numbered as 31, 32, 33, 43, 59, 77 and 79 were placed in the northern Italy, herds 82 and 84 in the centre of Italy and herd 57 in the south of Italy.

All the isolates included in the analysis were collected from 2007 to 2010 and derived from individual faecal samples by cultivation on Herrold's egg yolk medium (HEYM), containing 2 mg of mycobactin J/ml, supplemented with Chloramphenicol (30 mg/l) (HEYM/CAF) or with Nalidixic acid (50 mg/l), Vancomycin (50 mg/l) and sodium pyruvate (4 g/l) (HEYM/ANV). Suspected colonies were sub-cultured in HEYM/ANV medium with and without mycobactin. According to previous reports [10,11], Map identity was confirmed by Ziehl-Neelsen staining, mycobactin dependency, *IS900 *end-point PCR and by a newly developed F57 qPCR.

The DNA was extracted suspending one colony in 100 μl of distilled sterile water and boiling for 20 min. The bacterial lysate was directly used in PCR.

*IS900 *PCR reactions were carried out according to Taddei et al. [21]. F57-qPCR reaction was carried out on StepOne Plus system (Applied Biosystems, Monza, Italy), with 0.3 μM concentration for both primers (F57-forward ATAGCTTTCCTCTCCTTCGTC; F57-reverse CAGGGCAACAACATATTCGG). Cycle conditions were: initial denaturation/activation at 95°C for 10 min, followed by 35 cycles with 15 s of denaturation and 30 s annealing/extension at 62°C. PCR was monitored in real time by acquiring data with the SYBR Green channel (Ex 488 nm and Em 522 nm). The ***threshold cycles ***(Cts) were always lower than 30. The efficiency of PCR reaction (estimated as 96%) was calculated according to Pfaffl's method [22]. The analytical specificity of this PCR was tested using a panel of strains (derived from both culture collection and field isolates) belonging to *M. avium *subsp. *avium *(one strain and two field isolates), *M. avium *subsp. *silvaticum*, *M. avium *subsp. *hominissuis, M fortuitum*, *M gordonae*, *M. intracellulare*, *M. marinum*, *M. smegmatis*, *M. phley*, *M. scrofulaceum*, *M. microti*, *M. xenopi*, *M. bovis BCG*, *M. bovis *(two field isolates), *M. tuberculosis*, *M. terrae *and *M. porcinum *(three strains). No amplification was observed with these bacteria (data not shown).

### Assignment of Map-type

For the assignment to type S or C, we performed allele specific PCR (DMC-PCR) [23], while to exclude the presence of types B in our isolates, IS 1311 PCR, followed by restriction endonuclease analysis (PCR-REA), was done [24].

### Minisatellite typing

Ten loci were analysed according to the procedures reported in the original papers. Particularly, we amplified three loci (MIRU1, MIRU2 and MIRU3) described by Bull [6], two loci (VNTR1067 and VNTR3527) by Overduin [8] and five loci (VNTR25, VNTR47, VNTR3, VNTR7 and VNTR32) described by Thibault [9].

The number of repeats was calculated as reported in the guidelines for bacterial typing methods [20] after conventional gel electrophoresis (1.5% agarose gel).

### Microsatellite typing

Three microsatellite loci (SSR1, SSR2 and SSR8) were investigated. All PCR reactions were carried out in a final volume of 25 μl with @Taq (Euroclone, Pero, Italy) on "Mastercycler ep gradient s", according to Amonsin et al. [7]. The amplicons were sequenced by Beckman Coulter CEQ 8000 automated sequencer with DTCS Quick start chemistry (Beckman Coulter), according to the manufacturer's instructions. Raw traces were analysed and peaks were identified using "Sequencing " and "Investigator" packages of CEQ 8000 software (version 8.0). The number of repeats identified by the software was also checked by manual reading. During the amplification, repeated sequences can led to polymerase slippage, resulting in stutter peaks. These artefacts could induce to misleading interpretation of the number of nucleotide repeats present in the locus. To avoid any possible bias and according to a previous paper [12], we considered up to 11 residue repeats as suitable cut-off for the loci SSR1 and SSR2.

### Allelic Diversity and Simpson's Discriminatory Index

The Allelic Diversity (*h*) was calculated with the formula $1-\sum x_{i}^{2}\left(n∕n-1\right)$, where *n *is the number of isolates and *x_i _*is the frequency of the *i*th allele at the specific locus [25]. The Simpson's Index of Diversity (SID) was evaluated with the formula $1-\frac{1}{n(n-1)})\sum_{j=1}^{S}x_{j}(x_{j}-1)$, where *n *is the total number of isolates tested, S the number of different genotypes and *x_j _*is the number of isolates belonging to the *j*th genotype [26]. The index was calculated by using the free software Discriminatory Power Calculator http://insilico.ehu.es/mini_tools/discriminatory_power/, while confidence intervals of 95% were calculated according to Grundmann [27].

## Authors' contributions

MR conceived of the study, carried out the laboratory work, participated in its design, compiled and analysed data and drafted the manuscript. GB carried out part of the laboratory work (Minisatellites analysis) and analysed the data. RT helped to draft the manuscript and with EC carried out part of the laboratory work (Microsatellites analysis). GLB, GC and CAG participated in the conception and design of the study. NA participated and supervised the design of the study and helped to draft the manuscript. All authors read and approved the final manuscript.

## Supplementary Material

Additional file 1 **Table 1S: Number of isolates with the specific allele copy number and allelic diversity**. The file contain data on the allelic diversity of the loci considered in this study. Click here for file

Additional file 2 **Table 1S: MIRU-VNTR/SSR pattern of 98 Type C isolates coming from 10 herds**. The file contain data about the variability recovered inside 10 herds. Click here for file

## References

[B1] Stevenson K Alvarez J Bakker D Biet F de Juan L Denham S Dimareli Z Dohmann K Gerlach GF Heron I Kopecna M May L Pavlik I Sharp JM Thibault VC Willemsen P Zadoks RN Greig A Occurrence of Mycobacterium avium subspecies paratuberculosis across host species and European countries with evidence for transmission between wildlife and domestic ruminants BMC Microbiol 2009 9 212 10.1186/1471-2180-9-212 19811631 PMC2765967

[B2] Ott SL Wells SJ Wagner BA Herd-level economic losses associated with Johne's disease on US dairy operations Prev Vet Med 1999 40 179 192 10.1016/S0167-5877(99)00037-9 10423773

[B3] Abubakar I Myhill D Aliyu SH Hunter PR Detection of Mycobacterium avium subspecies paratuberculosis from patients with Crohn's disease using nucleic acid-based techniques: a systematic review and meta-analysis Inflamm Bowel Dis 2008 14 401 410 10.1002/ibd.20276 17886288

[B4] Motiwala AS Li L Kapur V Sreevatsan S Current understanding of the genetic diversity of Mycobacterium avium subsp. paratuberculosis Microbes Infect 2006 8 1406 1418 10.1016/j.micinf.2005.12.003 16697677

[B5] Ramel C Mini-and microsatellites Environ Health Perspect 1997 105 Suppl 4 781 9 10.1289/ehp.97105s4781 9255562 PMC1470042

[B6] Bull TJ Sidi-Boumedine K McMinn EJ Stevenson K Pickup R Hermon-Taylor J Mycobacterial interspersed repetitive units (MIRU) differentiate Mycobacterium avium subspecies paratuberculosis from other species of the Mycobacterium avium complex Mol Cell Probes 2003 17 157 164 10.1016/S0890-8508(03)00047-1 12944117

[B7] Amonsin A Li LL Zhang Q Bannantine JP Motiwala AS Sreevatsan S Kapur V Multilocus short sequence repeat sequencing approach for differentiating among Mycobacterium avium subsp. paratuberculosis strains J Clin Microbiol 2004 42 1694 1702 10.1128/JCM.42.4.1694-1702.2004 15071027 PMC387571

[B8] Overduin P Schouls L Roholl P van der Zanden A Mahmmod N Herrewegh A van Soolingen D Use of multilocus variable-number tandem-repeat analysis for typing Mycobacterium avium subsp. paratuberculosis J Clin Microbiol 2004 42 5022 5028 10.1128/JCM.42.11.5022-5028.2004 15528690 PMC525156

[B9] Thibault VC Grayon M Boschiroli ML Hubbans C Overduin P Stevenson K Gutierrez MC Supply P Biet F New variable-number tandem-repeat markers for typing Mycobacterium avium subsp. paratuberculosis and M. avium strains: comparison with IS900 and IS1245 restriction fragment length polymorphism typing J Clin Microbiol 2007 45 2404 2410 10.1128/JCM.00476-07 17537942 PMC1951273

[B10] Möbius P Luyven G Hotzel H Köhler H High genetic diversity among Mycobacterium avium subsp. paratuberculosis strains from German cattle herds shown by combination of IS900 restriction fragment length polymorphism analysis and mycobacterial interspersed repetitive unit-variable-number tandem-repeat typing J Clin Microbiol 2008 46 972 981 10.1128/JCM.01801-07 18174306 PMC2268378

[B11] El-Sayed A Hassan AA Natour S Abdulmawjood A Bülte M Wolter W Zschöck M Evaluation of three molecular methods of repetitive element loci for differentiation of Mycobacterium avium subsp. paratuberculosis (MAP) J Microbiol 2009 47 253 259 10.1007/s12275-008-0257-1 19557341

[B12] Thibault VC Grayon M Boschiroli ML Willery E Allix-Béguec C Stevenson K Biet F Supply P Combined multilocus short-sequence-repeat and mycobacterial interspersed repetitive unit-variable-number tandem-repeat typing of Mycobacterium avium subsp. paratuberculosis isolates J Clin Microbiol 2008 46 4091 4094 10.1128/JCM.01349-08 18923016 PMC2593263

[B13] Douarre PE Cashman W Buckley J Coffey A O'Mahony J Molecular characterization of Mycobacterium avium subsp. paratuberculosis using multi-locus short sequence repeat (MLSSR) and mycobacterial interspersed repetitive units-variable number tandem repeat (MIRU-VNTR) typing methods Vet Microbiol 2011 149 482 7 10.1016/j.vetmic.2010.12.001 21269784

[B14] Romano MI Amadio A Bigi F Klepp L Etchechoury I Llana MN Morsella C Paolicchi F Pavlik I Bartos M Leão SC Cataldi A Further analysis of VNTR and MIRU in the genome of Mycobacterium avium complex, and application to molecular epidemiology of isolates from South America Vet Microbiol 2005 110 221 237 10.1016/j.vetmic.2005.07.009 16171956

[B15] Castellanos E Romero B Rodríguez S de Juan L Bezos J Mateos A Domínguez L Aranaz A Molecular characterization of Mycobacterium avium subspecies paratuberculosis Types II and III isolates by a combination of MIRU-VNTR loci Vet Microbiol 2010 144 118 126 10.1016/j.vetmic.2009.12.028 20116185

[B16] Sevilla I Li L Amonsin A Garrido JM Geijo MV Kapur V Juste RA Comparative analysis of Mycobacterium avium subsp. paratuberculosis isolates from cattle, sheep and goats by short sequence repeat and pulsed-field gel electrophoresis typing BMC Microbiol 2008 8 204 10.1186/1471-2180-8-204 19032737 PMC2605457

[B17] Harris NB Payeur JB Kapur V Sreevatsan S Short-sequence-repeat analysis of Mycobacterium avium subsp. paratuberculosis and Mycobacterium avium subsp. avium isolates collected from animals throughout the United States reveals both stability of loci and extensive diversity J Clin Microbiol 2006 44 2970 2973 10.1128/JCM.00584-06 16891519 PMC1594651

[B18] Ricchi M Taddei R Barbieri I Belletti GL Pacciarini ML Arrigoni N Typing of Mycobacterium avium subsp. paratuberculosis (MAP) strains isolated from different Italian regions by four Variable-Number Tandem Repeat (VNTR) methods alone or in association Proceedings of 10th International Colloquium on Paratuberculosis 2009; Minneapolis (USA) 2009 60 63

[B19] van Hulzen KJ Heuven HC Nielen M Hoeboer J Santema WJ Koets AP Different Mycobacterium avium subsp. paratuberculosis MIRU-VNTR patterns coexist within cattle herds Vet Microbiol 2011 148 419 24 10.1016/j.vetmic.2010.09.029 21035277

[B20] van Belkum A Tassios PT Dijkshoorn L Haeggman S Cookson B Fry NK Fussing V Green J Feil E Gerner-Smidt P Brisse S Struelens M European Society of Clinical Microbiology and Infectious Diseases (ESCMID) Study Group on Epidemiological Markers (ESGEM) Guidelines for the validation and application of typing methods for use in bacterial epidemiology Clin Microbiol Infect 2007 Suppl 3 1 46 10.1111/j.1469-0691.2007.01786.x 17716294

[B21] Taddei R Barbieri I Pacciarini ML Fallacara F Belletti GL Arrigoni N Mycobacterium porcinum strains isolated from bovine bulk milk: implications for Mycobacterium avium subsp. paratuberculosis detection by PCR and culture Vet Microbiol 2008 130 338 347 10.1016/j.vetmic.2008.02.007 18378411

[B22] Pfaffl MW A new mathematical model for relative quantification in real-time RT-PCR Nucleic Acids Res 2001 29 e45 10.1093/nar/29.9.e45 11328886 PMC55695

[B23] Collins DM De Zoete M Cavaignac SM Mycobacterium avium subsp. paratuberculosis strains from cattle and sheep can be distinguished by a PCR test based on a novel DNA sequence difference J Clin Microbiol 2002 40 4760 4762 10.1128/JCM.40.12.4760-4762.2002 12454189 PMC154624

[B24] Whittington RJ Taragel CA Ottaway S Marsh I Seaman J Fridriksdottir V Molecular epidemiological confirmation and circumstances of occurrence of sheep (S) strains of Mycobacterium avium subsp. paratuberculosis in cases of paratuberculosis in cattle in Australia and sheep and cattle in Iceland Vet Microbiol 2001 79 311 322 10.1016/S0378-1135(00)00364-3 11267791

[B25] Mazars E Lesjean S Banuls AL Gilbert M Vincent V Gicquel B Tibayrenc M Locht C Supply P High-resolution minisatellite-based typing as a portable approach to global analysis of Mycobacterium tuberculosis molecular epidemiology Proc Natl Acad Sci USA 2001 98 1901 1906 10.1073/pnas.98.4.1901 11172048 PMC29354

[B26] Hunter PR Gaston MA Numerical index of the discriminatory ability of typing systems: an application of Simpson's index of diversity J Clin Microbiol 1988 26 2465 2466 3069867 10.1128/jcm.26.11.2465-2466.1988 PMC266921

[B27] Grundmann H Hori S Tanner G Determining confidence intervals when measuring genetic diversity and the discriminatory abilities of typing methods for microorganisms J Clin Microbiol 2001 39 4190 4192 10.1128/JCM.39.11.4190-4192.2001 11682558 PMC88515

