# Supplementary material for: Effectiveness of combination of Mini-and Microsatellite loci to sub-type Mycobacterium avium subsp. paratuberculosis Italian type C isolates
Source: BMC Vet Res. 2011 Sep 19;7:54. doi: 10.1186/1746-6148-7-54 (PMC3182896; doi:10.1186/1746-6148-7-54)
Supplement: Additional file 1 — Table 1S: Number of isolates with the specific allele copy number and allelic diversity. The file contain data on the allelic diversity of the loci considered in this study. [file 1746-6148-7-54-S1.DOC]

**Additional file 1, Table S1. Number of isolates with the specific allele copy number and allelic diversity.**

|  |  | **Number of isolates with the specific copy no.** | | | | | | | | | | | |  |
| --- | --- | --- | --- | --- | --- | --- | --- | --- | --- | --- | --- | --- | --- | --- |
|  | Locus | **1** | **2** | **3** | **4** | **5** | **6** | **7** | **8** | **9** | **10** | **11** | **>11** | Allelic diversity (*h*)^e^ |
| Mini | MIRU1^a^ |  |  | 84 |  |  |  |  |  |  |  |  |  | 0.00 |
|  | MIRU 2^a^ |  |  |  |  | 1 |  | 55 |  | 28 |  |  |  | 0.45 |
|  | MIRU 3^a^ |  |  | 1 |  | 83 |  |  |  |  |  |  |  | 0.01 |
|  | VNTR25^b^ |  | 1 | 83 |  |  |  |  |  |  |  |  |  | 0.01 |
|  | VNTR 32^b^ |  |  |  |  |  | 1 |  | 83 |  |  |  |  | 0.01 |
|  | VNTR 3^b^ |  | 84 |  |  |  |  |  |  |  |  |  |  | 0.00 |
|  | VNTR 7^b^ | 2 | 81 | 1 |  |  |  |  |  |  |  |  |  | 0.06 |
|  | VNTR 47^b^ |  | 1 | 83 |  |  |  |  |  |  |  |  |  | 0.01 |
|  | VNTR 1067^c^ | 2 | 78 | 4 |  |  |  |  |  |  |  |  |  | 0.12 |
|  | VNTR 3527^c^ | 13 | 71 |  |  |  |  |  |  |  |  |  |  | 0.25 |
| Micro | SSR1^d^ |  |  |  |  |  |  | 64 | 4 | 2 | 1 |  | 13 | 0.39 |
|  | SSR 2^d^ |  |  |  |  |  |  |  |  | 7 | 33 | 22 | 22 | 0.70 |
|  | SSR 8^d^ |  |  | 1 | 69 | 14 |  |  |  |  |  |  |  | 0.29 |

^a^ MIRU loci according to Bull et al. [6].

^b^ VNTR-MIRU according to Thibault et al. [9].

^c^ VNTR according di Overduin et al. [8].

^d^ SSR according to Amonsin et al. [7].

^e^ The allelic diversity was calculated as reported by Mazars et al. [24].
