# Supplementary material for: Effectiveness of combination of Mini-and Microsatellite loci to sub-type Mycobacterium avium subsp. paratuberculosis Italian type C isolates
Source: BMC Vet Res. 2011 Sep 19;7:54. doi: 10.1186/1746-6148-7-54 (PMC3182896; doi:10.1186/1746-6148-7-54)
Supplement: Additional file 2 — Table 1S: MIRU-VNTR/SSR pattern of 98 Type C isolates coming from 10 herds. The file contain data about the variability recovered inside 10 herds. [file 1746-6148-7-54-S2.DOC]

**Additional file 2 Table S2: MIRU-VNTR/SSR pattern of 98 Type C isolates coming from 10 herds.**

| **Herd No.^a^** | **Total strains** | **No. of strain for genotype** | **MIRU-VNTR/SSR**  **Type^b^** | **MIRU-VNTR**  **Type^c^** | **SSR**  **Type^d^** | **pattern^e^** |
| --- | --- | --- | --- | --- | --- | --- |
| 31 | 3 | 1 | MVS 24 | MV 10 | S 1 |  |
|  |  | 1 | MVS G1 | MV 5 | S 3 |  |
|  |  | 1 | MVS 31 | MV 10 | S 13 |  |
| 32 | 7 | 1 | MVS G2 | - | S 4 | 3,7,3,3,8,2,2,3,2,2,7,>11,4 |
|  |  | 2 | MVS G3 | MV 10 | - | 3,7,5,3,8,2,2,3,2,2,>11,9,5 |
|  |  | 1 | MVS 25 | MV 10 | S 2 |  |
|  |  | 1 | MVS 27 | MV 10 | S 4 |  |
|  |  | 2 | MVS 12 | MV 4 | S 11 |  |
| 33 | 50 | 10 | MVS 31 | MV 10 | S 13 |  |
|  |  | 6 | MVS G4 | MV 3 | S 4 | 3,9,5,3,8,2,2,3,2,2,7,>11,4 |
|  |  | 4 | MVS 30 | MV 10 | S 12 |  |
|  |  | 20 | MVS 32 | MV 10 | S 14 |  |
|  |  | 1 | MVS 4 | MV 3 | S 3 |  |
|  |  | 1 | MVS G5 | MV 8 | - | 3,7,5,3,8,2,2,3,1,2,>11,9,5 |
|  |  | 1 | MVS G6 | MV 10 | S 11 | 3,7,5,3,8,2,2,3,2,2,>11,10,4 |
|  |  | 4 | MVS 27 | MV 10 | S 4 |  |
|  |  | 1 | MVS G7 | MV 10 | S 15 | 3,7,5,3,8,2,2,3,2,2,>11,>11,4 |
|  |  | 1 | MVS 24 | MV 10 | S 1 |  |
|  |  | 1 | MVS G8 | MV 8 | S 13 | 3,7,5,3,8,2,2,3,1,2,>11,>11,5 |
| 43 | 3 | 2 | MVS 26 | MV 10 | S 3 |  |
|  |  | 1 | MVS 24 | MV 10 | S 1 |  |
| **57** | 3 | 3 | MVS 26 | MV 10 | S 3 |  |
| 59 | 3 | 2 | MVS 7 | MV 4 | S 3 |  |
|  |  | 1 | MVS 5 | MV 4 | S 1 |  |
| 77 | 14 | 14 | MVS 26 | MV 10 | S 3 |  |
| 79 | 5 | 3 | MVS 26 | MV 10 | S 3 |  |
|  |  | 2 | MVS 27 | MV 10 | S 4 |  |
| 82 | 7 | 4 | MVS 6 | MV 4 | S 2 |  |
|  |  | 2 | MVS 7 | MV 4 | S 3 |  |
|  |  | 1 | MVS 8 | MV 4 | S 4 |  |
| 84 | 3 | 3 | MVS 6 | MV 4 | S 2 |  |

**^a^** in bold and underlined are indicated farms from south and centre of Italy, respectively, while the rest of the farms are placed in the northern Italy. Herd no. corresponds to Herd no. in Table 3.

^b^ according to Table 3; G: new genotype not previously detected during the inter-herd analysis; the new profile is reported in the “pattern column”.

^c^ according to Table 1

^d^ according to Table 2

^e^ order: MIRU1, MIRU2, MIRU3, VNTR25,VNTR32, VNTR3, VNTR7, VNTR47, VNTR1067, VNTR3527, SSR1, SSR2, SSR8.
